# Facial expressions affect the memory of facial colors

**DOI:** 10.1167/jov.24.5.14

**Published:** 2024-05-30

**Authors:** Yuya Hasegawa, Hideki Tamura, Shigeki Nakauchi, Tetsuto Minami

**Affiliations:** 1 Department of Computer Science and Engineering, Toyohashi University of Technology, Toyohashi, Aichi, Japan

**Keywords:** facial expression, facial color, memory color

## Abstract

Facial color influences the perception of facial expressions, and emotional expressions bias how facial color is remembered. However, it remains unclear whether facial expressions affect daily facial color memory. The memory color effect demonstrates that knowledge about typical colors affects the perception of the actual color of given objects. To investigate the effect of facial color memory, we examined whether the memory color effect for faces varies depending on facial expression. We calculated the subjective achromatic point of the facial expression image stimulus and compared the degree to which it was shifted from the actual achromatic point between facial expression conditions. We hypothesized that if the memory of facial color is influenced by the facial expression color (e.g., anger is a warm color, fear is a cold color), then the subjective achromatic point would vary with facial expression. In Experiment 1, we recruited 13 participants who adjusted the color of facial expression stimuli (anger, neutral, and fear) and a banana stimulus to be achromatic. No significant differences in the subjective achromatic point between facial expressions were observed. Subsequently, we conducted Experiment 2 with 23 participants because Experiment 1 did not account for the sensitivity to color changes on the face; humans perceive greater color differences in faces than in non-faces. Participants selected which facial color they believed the expression stimulus appeared to be, choosing one of two options provided to them. The results indicated that the subjective achromatic points of anger and fear faces significantly shifted toward the opposite color direction compared with neutral faces in the brief presentation condition. This research suggests that the memory color of faces differs depending on facial expressions and supports the idea that the perception of emotional expressions can bias facial color memory.

## Introduction

Facial color is one of the factors used to judge facial expressions. A reddish face is more likely to be perceived as an angry face and is interpreted as having a higher emotional intensity of anger (Kato, Sato, & Mizokami, 2022; Minami, Nakajima, & Nakauchi, 2018; Nakajima, Minami, & Nakauchi, 2017; Thorstenson, Pazda, & Elliot, 2019; Thorstenson, Pazda, & Krumhuber, 2021). Nakajima et al. (2017) conducted an experiment in which participants responded to a set of facial stimuli where reddish-enhanced faces (CIE a* +12) morphed from fearful to angry expressions by indicating whether they perceived fear or anger. Reddish-colored faces were reportedly more likely to be perceived as angry. Similarly, Kato et al. (2022) investigated changes in anger perception when manipulating color related to hemoglobin and melanin for a set of face stimuli transitioning from neutral to angry expressions. They found that stimuli with increased hemoglobin concentration, which causes the face to appear reddish, and increased melanin concentration, which causes the face to appear yellowish, were more likely to be perceived as angry. Additionally, facial color affects perceived social characteristics (e.g., friendliness, aggression, health; Thorstenson & Pazda, 2021). A recent study focused on both explicit and implicit facial expressions (Nguyen et al., 2023). Nguyen et al. (2023) compared the friendliness ratings of reddish hybrid face stimuli in which the low-frequency component of the neutral facial expression image was replaced with the low-frequency component of the happy or angry facial expression. The results showed that reddish hybrid happy face stimuli were perceived as friendlier, suggesting that facial color modulates the perception of implicit facial expressions in hybrid facial stimuli. Furthermore, these effects were observed only for real faces, emoticons, and face models and were also enhanced by background color (Liao, Sakata, & Paramei, 2018; Minami et al., 2018; Qin, 2021; Thorstenson, McPhetres, Pazda, & Young, 2021), suggesting that specific colors enhance emotion perception and sensitivity to emotion discrimination. Additionally, even when controlling for color change and the number of emotions and colors, Peromaa and Olkkonen (2019) reported that red affected the perception of an angry face, albeit to a small extent (Peromaa & Olkkonen, 2019). Thus, do humans really remember the color of angry faces as being redder than neutral faces?

The criteria for judging human complexion are thought to be based on skin color and conditions that humans typically remember (Hasantash, Lafer-Sousa, Afraz, & Conway, 2019; Shimakura & Sakata, 2022). The relationship between color and emotion is known to influence human facial color memory. For example, Thorstenson, Pazda et al. (2021) conducted a facial color recall task after presenting facial expression stimuli. Consequently, the redder and yellower (CIE a*+, b*+) face was chosen in the recall task significantly more often than the face that was actually displayed, suggesting that the remembered facial color was biased toward red and yellow. They also noted that blushing (e.g., anger) was recalled more vividly than paling (e.g., fear) for the red-yellow component of facial color. Thus, at least in temporal memory during the experimental period, the facial color components were amplified in memory, and their magnitude changed depending on facial expression.

However, previous studies have focused only on temporary color memories, and no study has yet investigated whether such memory biases are empirically driven in daily life (e.g., Thorstenson, Pazda et al., 2021). If facial expressions bias temporary color memory retention, a similar bias is likely to appear in long-term memory. Therefore, humans may remember the facial color of certain expressions more vividly in the long term, forming social and experiential memories related to their daily lives. Thus, we focused on the color of objects related to experiential, long-term memory from the perspective of color memory.

Memory color is defined as the typical color of an object that a human acquires through their experience (Witzel & Gegenfurtner, 2013). For example, most people recognize that the color of a ripe banana is yellow; this knowledge of typical colors is the memory color. Memory color influences human color perception (Hansen, Olkkonen, Walter, & Gegenfurtner, 2006; Olkkonen, Hansen, & Gegenfurtner, 2008). Hansen et al. (2006) conducted a color adjustment experiment in which the color of fruit and vegetable stimuli presented on display was adjusted to an achromatic or typical color. They found that the achromatic adjustment points of the stimulus shifted in the opposite color direction to the typical color, which was the color of the object memorized by the participant, and the fruit that was physically achromatic in the color space appeared to be colored in the typical color (Olkkonen et al., 2008). As another example of investigating the effect of memory color, Witzel conducted online experiments in which participants were asked to rate the colors of two stimuli. The results showed that even in the absence of strict color calibration, participants were significantly more likely to select a bluish banana over a pure gray banana, suggesting robust color perception changes because of the memory color effect (Witzel, 2016). From these previous studies, we combined the idea that the memory color paradigm could be used to reveal memorized colors associated with facial expressions through long-term memory.

Therefore, this study aimed to clarify the dependence of facial color memory on facial expressions, specifically focusing on the effect of memory color. The relationship between facial color and facial expression raises the following research question: Do humans remember angry faces as reddish rather than neutral? Due to objects like bananas, which have a typical color, the achromatic state appears tinted in memory color, and the subjective achromatic point shifts in the opposite color direction. For instance, if an observer memorizes an angry face as more reddish-yellowish than neutral in one's life experience, the red and yellow components of the memory color for angry faces will have higher saturation than for neutral faces. Thus, we hypothesized that the subjective achromatic point is more likely to shift toward the opposite color direction of the facial tone. In other words, we can anticipate evaluating the differences in the memory color of each facial expression based on the magnitude of the shift from the participants’ subjective achromatic point of each facial expression when the memory color of a neutral face is used as a reference. Building upon previous research (Hansen et al., 2006; Olkkonen et al., 2008), Experiment 1 attempted to estimate the effect of memory color using an adjustment task. In Experiment 2, we estimated the effect of memory color using the constant method, referencing previous research (Nakajima et al., 2017).

## Experiment 1

First, we conducted a color adjustment task to estimate differences in memory color from the subjective achromatic point of each facial expression, following the methods outlined by Hansen et al. (2006) and Olkkonen et al. (2008).

### Participants

The number of participants was determined to be 15 based on a previous study (Olkkonen et al., 2008). Owing to the experiment's duration, we were able to recruit 13 students from Toyohashi University of Technology (two women, mean age 22.46 ± 0.93) to participate. We used the Ishihara Color Vision Test Chart II Concise Version 14 Table, provided by the Public Interest Incorporated Foundation Isshinkai, Handaya Co., Ltd, Tokyo, Japan. All participants correctly matched the prepared answers. They were provided with an introduction to the experiment, excluding the study's hypothesis (see Procedure and task), and gave informed consent to participate. This experiment was conducted with the approval of the Ethics Committee for Human Research at Toyohashi University of Technology and adhered strictly to the approved guidelines of the committee and the Declaration of Helsinki. This study was not preregistered.

### Stimuli

**Figure 1.**
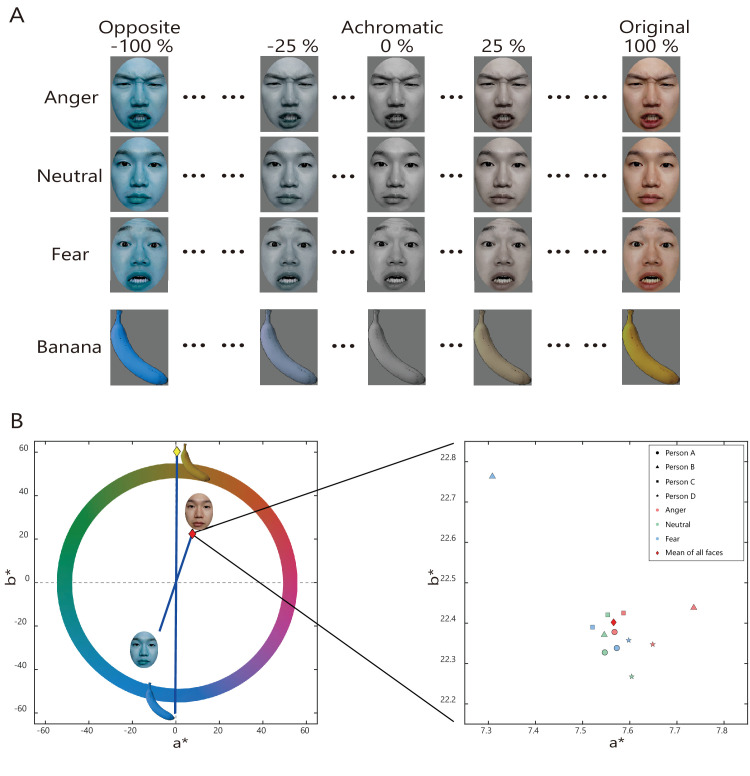
(**A**) Examples of the color state for each image condition. (**B**) The mean a*-b* values of the image stimuli. A red diamond represents the mean of all face image stimuli, and a yellow diamond represents the mean of the banana stimulus. Blue lines denote the averages of color change axes for the face and banana stimuli. Individual points on the right side represent each face image stimulus, including three facial expressions and four individuals. The red diamond corresponds to the left side. The faces in the figure are one of the author's faces (Y.H.), which was not used in the experiment.

Twelve facial images were prepared as stimuli (Figure 1A), which were obtained from angry, neutral, and fearful faces of four Japanese individuals (two women and two men) from the ATR Facial Expression Image Database (ATR-Promotions, Kyoto, Japan, https://www.atr-p.com/products/face-db.html). Previous research (Nakajima et al., 2017) compared anger and fear using the same database of facial images. We used angry and fearful faces to compare with the baseline (neutral face) because the color directions associated with angry and fearful faces were different (Thorstenson, Elliot, Pazda, Perrett, & Xiao, 2018). The hair, ears, and neck in the images were removed in an oval shape using Photoshop (Adobe Systems Inc., San Jose, CA, USA). Additionally, a banana image was used as a control condition, as in the memory color measurement experiment (Nakajima, Minam, & Nakauchi, 2010).

All images were adjusted to maintain an average image luminance of 16.9 cd/m^2^ using “SHINE_color,” a MATLAB 2021a toolbox (Mathworks, Natick, USA) (Ben, 2021; Willenbockel et al., 2010). The image dimensions were 4.3° × 5.7°. The mean and standard deviation of CIE L*a*b* values in the original faces were 47.63 ± 0.05 in L*, 7.57 ± 0.10 in a*, and 22.4 ± 0.12 in b*, respectively (See Figure 1B). The background color was gray, with a luminance identical to the average image luminance ([x,y] = =0.31, 0.33], Y = 16.9 cd/m^2^).

### Apparatus

The experiment was conducted in a booth, with stimuli presented on a monitor (EIZO CG319X; Eizo Corporation, Hakusan, Ishikawa, Japan; Resolution: 1920 × 1080; frame rate: 60 Hz) set at 200 lx in front of the participant's chair. The monitor was calibrated using a spectroradiometer (SR-3AR; Topcon, Tokyo, Japan), with its white-point chromaticity was *x* = 0.31, *y* = 0.33, *Y* = 99.7 cd/m^2^. The participants were seated and performed the task while keeping their heads on a chin rest positioned 70 cm away from the display. Psychotoolbox 3.0.17 served as the experimental control software (Brainard, 1997; Kleiner et al., 2007; Pelli, 1997).

### Procedure and task

**Figure 2.**
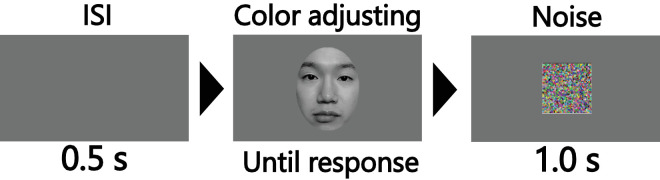
Procedure of Experiment 1. Notably, the ratio of the screen to the stimulus depicted in this figure differs from the actual ratio.

Figure 2 depicts a summary of the experimental procedure. Following a 0.5-second inter-stimulus interval, participants adjusted the color of the presented stimulus to achromatic using a numeric keypad. They were instructed as follows: “Please adjust the color of the presented image stimulus to achromatic using two buttons. Each button corresponds to a typical color and its opposite color direction.” We used the CIE L*a*b* space, which has been used in several prior studies to render faces reddish by modulating the a* value for controlling the colors of the stimuli (Minami et al., 2018; Nakajima et al., 2017; Thorstenson et al., 2018; Thorstenson, Pazda et al., 2021). Additionally, it was possible to alter the color while maintaining lightness. The conversion from RGB values to L*a*b* values in MATLAB was based on the XYZ values of D65, because the display was calibrated close to D65. The colors of the stimuli varied linearly, connecting the actual image color point in the L*a*b* space to the achromatic point.

Participants manipulated the stimulus color in the original-opposite direction (Figure 1B). Since the image information varies depending on each image, the original-opposite color directions were set accordingly. The two buttons on the numeric keypad corresponded to the color directions of the original and opposite colors, respectively, and one press changed the state to $\alpha =\tan(\frac{\pi }{4}\beta )\%$, where α represented the state of the stimulus color and β was a variable with increments or decrements of 1%. When the achromatic color was 0%, the original color was 100%, and the opposite color was −100%; thus the amount of color change by the numeric keypad was nonlinear. This nonlinear manipulation function was determined by a preliminary experiment in which the participants found it easy to operate.

When the participant pressed the button indicating they had finished adjusting, a Mondrian noise image (width 13.8° × height 13.8°) was presented for 1.0 second to eliminate the image aftereffects. The stimuli were presented five times per image in a random order, beginning with a randomly selected initial color near achromatic (β = −10 ∼ 10%). There were 65 trials in total (13 images × 5 repetitions). The participant was allowed to take a break every 13 trials, and the duration of the break was left to the participant's discretion.

### Data analysis

The achromatic adjustment values judged by the participants as achromatic were averaged for each condition (anger, neutral, fear, and banana). Then, we calculated the degree to which the achromatic adjustment point shifted as a percentage of the original/opposite color for each condition across all participants. We conducted a Friedman test on the facial expression condition using R because the normality of the data was not confirmed by the Shapiro–Wilk test. Additionally, for the banana condition, since the normality of the data was confirmed by the Shapiro–Wilk test, a one-sample *t*-test was performed to examine whether we replicated the effect of memory color, as suggested by a previous study (Olkkonen et al., 2008).

### Results and discussion

**Figure 3.**
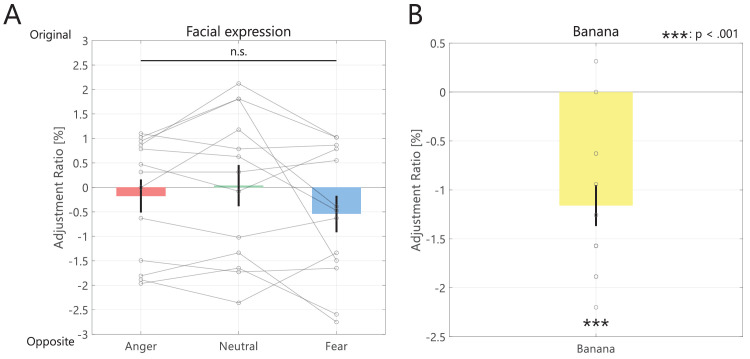
Results of Experiment 1. Participants’ mean of the achromatic adjustment point (**A**, Facial expression condition; **B**, Banana condition). Each gray points show individual data. Error bars represent the standard error of the mean.

Figure 3A displays the adjusted results for all facial expressions. The average adjusted points and their standard errors were −0.18 ± 0.34, 0.04 ± 0.42, −0.54 ± 0.37 for anger, neutral, and fear conditions, respectively. There was no significant main effect of facial expression condition (χ^2^ = 0.154, *p* = 0.926). This suggests that the memory color of a face does not depend on the facial expression. One possible factor was the prolonged presentation of the image stimulus. The adjustment task took an average of 9.39 seconds (with a maximum of 48.80 seconds), suggesting that chromatic adaptation may have extended the achromatic perceptual range (Oleari, 2016). In addition, the correlations in each facial expression condition were calculated from the achromatic adjusted value and response times of all trials. As a result, weak positive correlations were found between the adjustment value and reaction time for the facial expression conditions (Anger: n = 260, *p* = 0.190, adj. *p* < 0.001; Neutral: n = 260, ρ = 0.155, adj. *p* < 0.01; Fear: n = 260, *ρ* = 0.289, adj. *p* < 0.001). The longer the task time, the greater the adjustment value tended to be in the original color direction. Furthermore, humans are thought to be more sensitive to color changes in faces than in non-faces. Humans perceive a larger color difference in faces than in non-faces, even with the same amount of color differences (Thorstenson, Pazda, & Elliot, 2017). Therefore a better method might involve calculating the memory color effect in faces from the observer's color discrimination to several facial color stimuli rather than from a procedure in which observers can perceive continuous color changes.

Contrary to this, in Figure 3B, the achromatic adjustment ratio of the banana (−1.16 ± 0.21) significantly shifted in the opposite color direction (*t*[12] = −5.561, *p* < 0.002, Cohen's *d* = −1.542). This finding suggests that the banana is shifted more in the opposite color direction than the neutral gray and supports previous studies reporting that the achromatic adjustment point of bananas was farther than the subjective achromatic adjustment point (defined as the achromatic adjustment point of a control image such as a noise disk) in the opposite color direction (Hansen et al., 2006; Olkkonen et al., 2008). Our results are similar to those of previous research, where bananas with slightly opposite colors (slightly bluish) were judged to be grayer than bananas with the same chromaticity as the background color (Witzel, 2016).

In summary, in Experiment 1, we conducted a color-adjustment task in which participants adjusted the stimulus color to make it achromatic. However, the results showed no significant differences in the memory color effect of facial expressions. A potential reason for these findings could be that chromatic adaptation may be caused by prolonged presentation, and humans are thought to be more sensitive to color changes in faces. Thus, Experiment 2 investigated differences in memory color effects between facial expressions based on a task in which the presentation time was shortened and the colors of stimuli were selected from two color choices. We refer to the simpler color selection task by Witzel (2016).

## Experiment 2

In the hypothesis, if the memory of facial color is affected by facial expression color (e.g., anger being red, fear being blue), the subjective achromatic point of the face would vary depending on the facial expression. However, the results of Experiment 1 showed no significant difference in subjective achromatic points between facial expressions. A possible explanation for these results could be the occurrence of chromatic adaptation, which may have expanded the range of achromatic perception, resulting in large individual differences in subjective achromatic points in the facial expression condition. Therefore, in Experiment 2, a color selection task was conducted using a “Yes/No task” in which participants selected the briefly presented image stimulus that they thought appeared to be a certain color. This method was used to calculate the subjective achromatic point and to investigate differences in facial color memory between facial expressions.

### Participants

Twenty-three students (four women, mean age 20.87 ± 1.32) participated in this study at Toyohashi University of Technology. The sample size was calculated using PANGEA (Westfall, Kenny, & Judd, 2014) with an effect size of *d* = 0.5, power = 0.8, and α = 0.05. As in Experiment 1, all participants were fully informed about the experiment and consented to participate; only participants who passed the color vision test were recruited.

### Stimuli

**Figure 4.**
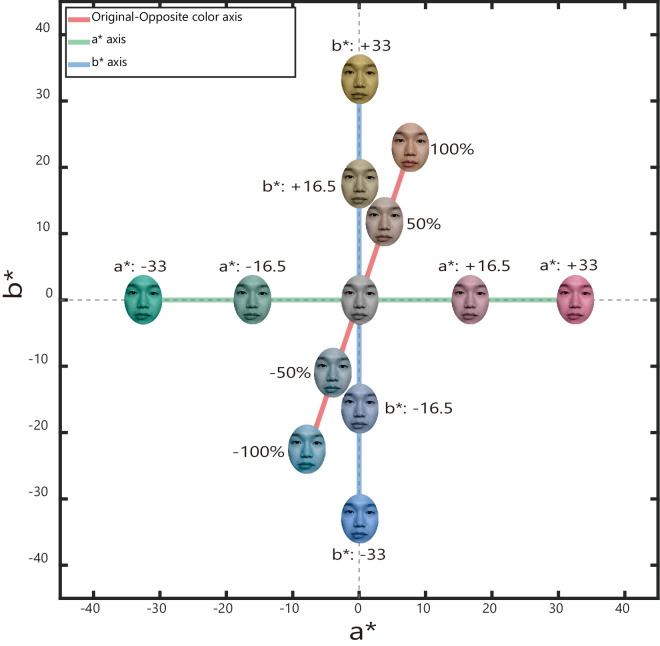
Each axis of condition in Experiment 2. The original-opposite color axis in the figure is the average line of all the faces.

The facial expression stimuli and their color change between the point of the actual image color and its achromatic counterpart in L*a*b* (original-opposite axis) were consistent with Experiment 1. Additionally, to confirm whether the angry face simply appears reddish visually rather than as a result of a memory color effect, we included the axes of a* (red-green) and b* (yellow-blue) in the CIE L*a*b* space as control conditions (see Figure 4). We used these conditions to confirm whether the achromatic angry and fearful faces simply appear reddish/yellowish and greenish/blueish visually, respectively, rather than exhibiting a memory color effect. We hypothesized that the subjective achromatic points of faces are not different between facial expressions in a* and b* axis conditions because the angry and fearful faces did not affect facial color judgment in previous research (Nakajima et al., 2017). In the original-opposite condition, the face stimuli changed color within a range of −4.5% to 4.5% in 1.5% increments (seven steps), with the defined states being achromatic: 0%, original: 100%, and opposite color: −100%. Moreover, in a* and b* conditions, the face stimuli changed color within a range of −1.50 unit to 1.5 in 0.5 increments (seven steps) for each condition. These ranges and step widths were determined based on preliminary experiments.

### Apparatus

The experimental environment was the same as that of Experiment 1.

### Procedure and task

**Figure 5.**
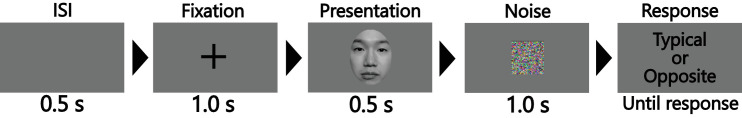
Procedure of Experiment 2.

Figure 5 illustrates the procedure used in Experiment 2. After a 0.5 second inter-stimulus interval and 1.0 second fixation were presented, the participants viewed a facial stimulus for 0.5 second. Then, the presentation of noise for 1.0 second to reduce the aftereffect. According to the instructions, the participant rated the color appearance of the presented facial stimulus using a numeric keypad (e.g., ``4'' opposite, ``6'' typical, in the original-opposite condition). The participants were instructed to respond intuitively, prioritizing accuracy.

The choices were modified based on the conditions, such as “red” or “green” in condition a*, and “yellow” or “blue” in condition b*. There were 252 facial stimuli (4 individuals × 3 expressions [anger, neutral, fear] × 7 steps × 3 axis conditions), and each stimulus was assessed twice. Therefore, each participant completed 504 trials, which were divided into three blocks per axis condition with a counterbalanced block order among participants. Each block was subdivided into four sections, and participants could take breaks between sections and blocks.

### Data analysis

Because of poor performance, we excluded participants whose selected facial color rates in conditions with minimum and maximum color changes were outside the plus and minus three sigma ranges of the participants’ averages, respectively. Thus, two participants were excluded from the analysis, leaving 21 for further analysis. These participants might have found it difficult to judge color differences (which cannot be assessed in the color vision test chart) and may have deviated from the range we set based on the preliminary experiment.

In each condition, the facial color selection rates for each facial expression from each participant were fitted with a psychometric function using a generalized linear model with a binomial distribution in the MATLAB Palamedes Toolbox (Prins, 2013; Prins, 2023; Prins & Kingdom, 2018). The point of subjective equality (PSE) was computed, which is shown as the subjective achromatic point. We checked the normality of the data using the Shapiro-Wilk test. If the data were confirmed to be normal, we performed a repeated measure one-way analysis of variance using the statistical tool Anovakun version 4.8.5 in *R*. If the data did not follow a normal distribution, we performed a Friedman test. When a significant main effect on facial expression conditions was found by the Friedman test, we performed post hoc tests using the Wilcoxon test. The *p* values were adjusted using the Shaffer method in the post hoc tests.

### Results and discussion

**Figure 6.**
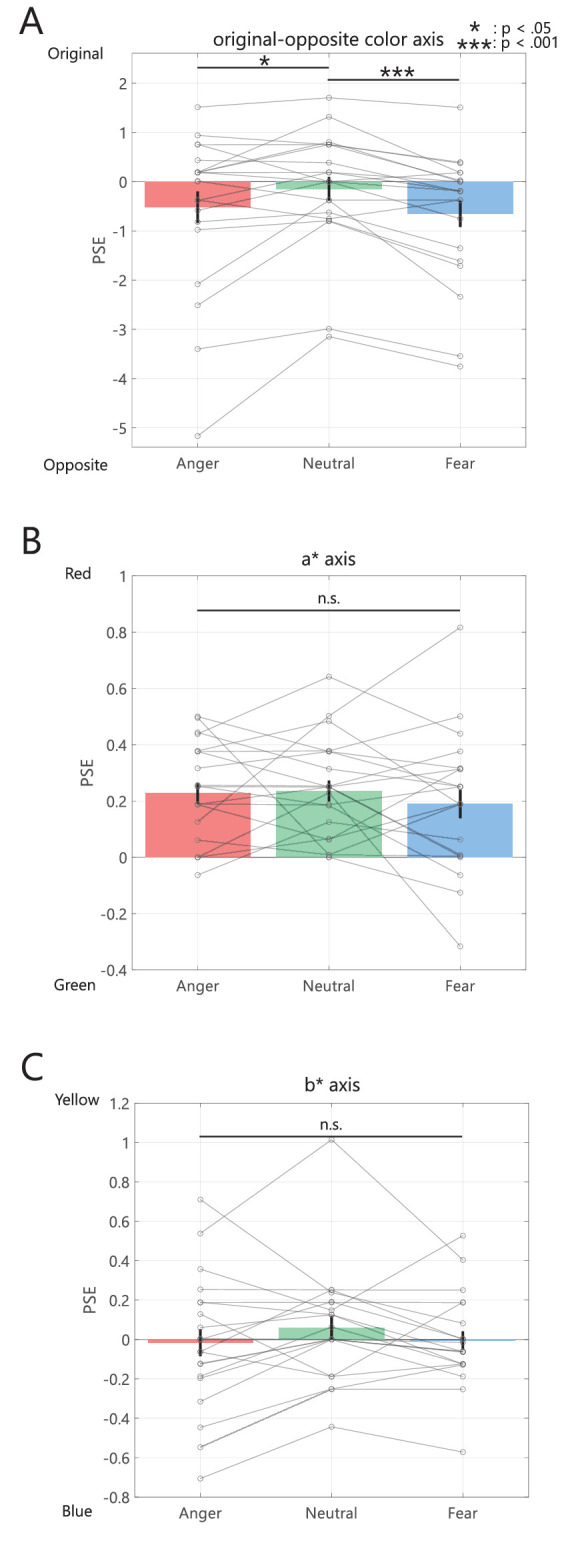
Results of Experiment 2. Participants mean of PSE ([**A**] original-opposite color axis, [**B**] a* axis, [**C**] b* axis). The closer the value is to “0,” the closer the subjective achromatic point is to the physical achromatic point. A larger positive value indicates that the subjective achromatic point is more shifted toward the original color (**A**)/red (**B**)/yellow (**C**), whereas a larger negative value indicates that the subjective achromatic point is more shifted toward the opposite color (**A**)/green (**B**)/blue (**C**). The other formats were the same as in Figure 3.

Figure 6 shows the mean PSE for each facial expression. We found significant differences between facial expressions in the original-opposite condition (χ^2^ = 14, *p* < 0.001) as illustrated in Figure 6A. The post hoc test revealed that the subjective achromatic points of anger and fear were shifted more toward opposite colors than toward neutral faces: anger-neutral (Z[20] = −2.07, adj. *p* < 0.05, *r* = 0.451) and fear-neutral (*Z*[20] = −3.53, adj. *p* < 0.001, *r* = 0.770). These findings suggest that participants perceived physically achromatic anger and fear faces as having a more typical color (skin tone) appearance compared to a neutral face. The memory colors of anger and fear faces exhibited a stronger skin tone component (higher saturation of red and yellow) compared to neutral faces.

In contrast, there were no significant differences between facial expressions in the a* and b* conditions in Figures 6B and 6C, respectively (a* condition: *F*[2,40] = 0.60, *p* = 0.556, ƞ_p_^2^ = 0.029), (b* condition: χ^2^ = 5.29, *p* = 0.071). These results show that angry faces do not necessarily appear reddish compared to other facial expressions and suggest that the memory color of the face depends on facial expression.

## General discussion

In this study, we estimated the effect of memory color on different facial expressions. To investigate this research question, we conducted the color adjustment task (Experiment 1) and the yes/no task (Experiment 2), comparing participants’ subjective achromatic points of angry, neutral, and fearful faces. The results of Experiment 1 showed no significant differences between facial expressions in the color-adjusting task (Figure 3A). The results of Experiment 2 showed that the subjective achromatic points of anger and fear shifted more towards the opposite color than in the neutral condition (Figure 5A). This result is similar to that of previous studies on the memory color effect, wherein objects in a physically achromatic state appear to be colored in their memory color, and the subjective achromatic point shifts to the opposite (Hansen et al., 2006; Olkkonen et al., 2008). Therefore, the angry and fearful faces were shown to be more likely to cause color misperception than neutral faces because of the memory color effect, and it is suggested that the memory color of angry and fearful faces has stronger facial color components, such as reddish-yellowish (high saturation), than neutral. In addition, our result is also similar to the results of a previous study, which demonstrated stronger memorization/recall of the red-yellow (a*+ and b*+) components than of the actual facial color, supporting the idea that the perception of emotional expressions can bias facial color memory (Thorstenson, Pazda et al., 2021).

The subjective achromatic point shifted to the opposite color in anger, supporting our hypothesis and aligning with the results of previous studies indicating that angry faces with a red-yellow component are more strongly memorized (Thorstenson, Pazda et al., 2021). Although fear is typically associated with decreased redness and yellowness (Thorstenson et al., 2018), the subjective achromatic point of a fearful face was also shifted towards the opposite color, similar to anger. Another previous study reported that the perceived intensity of fear was enhanced not only in the green direction (a−) but also in the red direction (a+; Thorstenson, McPhetres et al., 2021). Thus, it is possible to conclude that fearful faces evoke warm color effects, as well as cold color effects, and these warm color effects may manifest as memory color effects.

In the a* axis condition of Experiment 2 (Figure 5B), all subjective achromatic points tended to shift more toward the red (a*+ ) direction than toward the physical achromatic point (one-sample *t*-test, anger: *t*[20] = 5.78, *p* < 0.02, Cohen's *d* = 0.29; neutral: *t*[20] = 5.97, *p* < 0.001, Cohen's *d* = 0.30; fear: *t*[20] = 3.51, *p* < 0.05, Cohen's *d* = 0.25).One possible reason for this is that the achromatic face may have been perceived as having a morbid complexion because the facial color of the stimuli was not realistic and had little red or yellow color (Hasantash et al., 2019; Stephen, Law Smith, Stirrat, & Perrett, 2009). Facial color plays an important role not only in emotions but also in attractiveness and health status (Thorstenson et al., 2019; Thorstenson & Pazda, 2021). Specifically, a pale face is difficult to consider healthy, and face color, in the presence of impaired retinal mechanisms for color, such as the scenes under low-pressure sodium light, appears to have a morbid greenish complexion (Hasantash et al., 2019; Stephen et al., 2009; Thorstenson et al., 2019). Thus, participants were more likely to perceive the face color in the physically achromatic state as greenish when judging reddish or greenish.

Facial expressions in the color adjustment experiment (Experiment 1) did not differ but did significantly differ in the color selection experiment (Experiment 2). One possible reason for this discrepancy is the differences in the presentation time and perception of color change between the two experiments. In Experiment 1, the participants had to constantly observe the stimuli to adjust their color to achromatic conditions and visualize the color change. Humans evaluate color differences in facial stimuli to a greater extent than in non-facial stimuli, suggesting that humans are more sensitive to changes in facial colors (Thorstenson et al., 2017). Additionally, Experiment 1 provided a paradigm in which near-achromatic colors with the same average luminance as the background color were always observed, which may have caused chromatic adaptation even though we did not test for it. Thus, future studies should note continuous color changes and prolonged presentations when testing the effects of memory color on faces.

This study has some limitations that should be addressed in future research. First, there may be differences between the facial expressions defined by the database and those subjectively recognized by the participants. The stimuli were obtained from the ATR Facial Expression Image Database, and the classification of facial expressions was based on the emotional ratings associated with the data. Participants in this study did not classify the perceived facial expressions. For instance, if fear is perceived as a surprise, the effect of color on facial expression may have been in the opposite direction to that of a fearful face, which is expected to respond to cold colors (e.g., blue), as opposed to warm colors (e.g., red; Thorstenson et al., 2018).

Second, the results may have been influenced by cultural factors. Memory colors are shaped by individuals’ personal experiences. Therefore, only participants familiar with Japanese culture were included in the experiments because the stimuli consisted of Japanese faces. Given that it is recognized that color preferences for emotions and complexions can vary across cultures, it is reasonable to assume that the findings of this study reflect a restricted scope of occurrences (Han et al., 2018; Jonauskaite et al., 2020; Liao et al., 2018).

Third, we tested only angry and fearful faces as emotional expressions. Our results suggest that facial color, when combined with facial expressions, is retained as a more saturated memory color than neutral faces, using the same stimuli as in Nakajima et al. (2017). However, it is unclear what the results for other facial expressions would be, especially those such as “sad,” in which the color blue is associated with words and emoticons (Liao et al., 2018; Takahashi & Kawabata, 2018). Nakajima et al. reported that sad faces tended to appear more bluish than neutral or happy faces in their experiment to judge facial color (Nakajima et al., 2017). However, Thorstenson et al. reported that even paling emotions, such as fear and sadness, were more strongly memorized/recalled than the original images, although to a smaller degree than blushing emotions, such as anger and happiness (Thorstenson, Pazda et al., 2021). Thus, it is necessary to examine in future works whether color memorization changes depending on the presence of facial expressions or the semantic color of emotions, given the reported effects on colors in different directions in color spaces such as CIE L*a*b*.

Fourth, we did not individually research the characteristics of the participants. Abilities in the face and facial expression recognition, as well as attention biases towards specific emotions, are known to differ depending on trait anxiety (Surcinelli, Codispoti, Montebarocci, Rossi, & Baldaro, 2006; Telzer et al., 2008). Additionally, symptoms such as autism spectrum disorder and Moebius syndrome have been reported to potentially decrease efficiency in processing facial expressions (Golarai, Grill-Spector, & Reiss, 2006; Quettier, Maffei, Gambarota, Ferrari, & Sessa, 2023; Tanaka & Sung, 2016). Therefore differences in facial expression perception due to individual characteristics may have affected the memory color effect of facial expressions.

## Conclusions

We investigated whether the memory color effect differs depending on facial expression, based on the hypothesis that the memory color of an angry face is more saturated and the subjective achromatic point is more likely to shift in the opposite color direction than that of a neutral face. The results showed that the subjective achromatic points for anger and fear were shifted in the opposite color direction, suggesting that humans retain the memory color of emotional faces differs from neutral faces, and their colors are more reddish and yellowish than neutral faces. Our findings support the idea that facial expressions affect human facial color memory.
